# Affective disorder associated with post-traumatic epilepsy, misdiagnosis and under treatment: A case report

**DOI:** 10.1192/j.eurpsy.2022.1147

**Published:** 2022-09-01

**Authors:** E. Giourou, A. Theodoropoulou, S. Yfantis, O. Prodromaki, E. Georgila, P. Gourzis

**Affiliations:** General University Hospital of Patras, Greece, Department Of Psychiatry, Patras, Greece

**Keywords:** epilepsy, TBI, carbamazepine, affective disorders

## Abstract

**Introduction:**

A history of traumatic brain injury (TBI) is often associated with acquired epilepsy, which is associated with psychiatric co-morbidity, that when undetected might lead to misdiagnosis and mistreatment.

**Objectives:**

The objective is to present the case of a 47-years-old male with a history of TBI and undetected acquired epilepsy, with a subsequent treatment resident mood disorder that was lead to a full clinical remission once epileptic activity was controlled using anti-seizure monotherapy.

**Methods:**

After compulsory admittion to our inpatient psychiatric unit because of suicidal ideation and persistent aggressive behavior with volatile mood swings, the patient was fully evaluated and his psychiatric and medical histories were recorded. A brain CT scan and EEG were performed. Laboratory tests excluded other medical co-morbidity.

**Results:**

The patient had a previous history of TBI and subsequent multiple episodes of mood disorders that failed to reach full remission even if treated with antidepressives and antipsychotics for adequate time and dosage according to current quidelines. EEG was positive for epileptiform activity with sporadic slow theta waves and right frontotemporal epileptic-like features while the patient was free of clinical seizures. Carbamazepine was initiated and titrated up to 1200mg daily leading to the full remission of the initial clinical symptoms along with the EEG findings’ improvement. The patient remained stable with his functionality at its utmost recovery during the two-years follow-up evaluations.

**Conclusions:**

TBI induced epilepsy might be under-diagnosed in the absence of clinical seizures leading to the mistreatment of the associated psychiatric disorders that could be the only clinical presentation of the underlying pathology.

**Disclosure:**

No significant relationships.

